# Editorial: Decoding vascular aging: unraveling the enigma of pathological conditions and pre-mature vascular aging

**DOI:** 10.3389/fphys.2025.1707757

**Published:** 2025-10-02

**Authors:** Mitra Esfandiarei, Roshanak Rahimian

**Affiliations:** ^1^ Biomedical Sciences Program, College of Graduate Studies, Midwestern University, Glendale, AZ, United States; ^2^ Department of Basic Medical Sciences, College of Medicine, University of Arizona, Phoenix, AZ, United States; ^3^ Department of Anesthesiology, Pharmacology, and Therapeutics, Faculty of Medicine, University of British Columbia, Vancouver, BC, Canada; ^4^ Department of Pharmaceutical Sciences, Thomas J. Long School of Pharmacy, University of the Pacific, Stockton, CA, United States

**Keywords:** vascualr aging, vascular disease, brian pathology, neurovascular disease biomarkers, pre-mature aging

## Introduction

Vascular aging represents a fundamental biological process and a key determinant of cardiovascular morbidity and mortality. Far from being solely determined by chronological age, process such as smooth muscle phenotypical switches, endothelial mechanotransduction, mitochondrial quality control, and immune cells response together with organ and tissue level mechanisms such as neurovascular coupling, blood–brain barrier integrity, and life-course exposures such as diet, sleep, and infection. The contributions to this Research Topic bridge basic, translational, and clinical sciences, offering novel perspectives on mechanisms underlying normal and premature vascular aging, emerging risk factors, and potential therapeutic strategies.

In this Research Topic, Kennedy et al. highlight how endothelial cells translate hemodynamic forces into genomic programs—and how this “language of flow” can be distorted in Hutchinson Gilford Progeria Syndrome (HGPS). They demonstrate that endothelial cells elongate poorly under laminar flow and mount a blunted, misdirected transcriptional response in which a cholesterol-homeostasis program and the athero-inflammatory lectin LGALS3 are aberrantly upregulated. Importantly, correction of upstream nuclear-lamina defect restores elements of the shear-stress response, thereby tying genotype to a failure of flow protection.

In parallel, Wendt et al. have shown that short *ex vivo* exposure of mouse thoracic aortas to oxidized low-density lipoprotein (oxLDL) reprograms vasoreactivity through lectin-like oxLDL receptor 1 (LOX-1) in an endothelium-dependent manner, striking age- and sex-specific differences in stiffness, diameter, and vascular remodeling. The observed effects were attenuated by receptor inhibition. Collectively, these findings suggest that pro-aging inputs—nuclear-lamina pathology and oxidized lipids—disrupt endothelial sensing of both mechanical and chemical cues, thereby predisposing vessels toward dysfunction before structural disease becomes apparent.

Mitochondrial pathways surface repeatedly as both drivers and sensors—of aging phenotypes. Luo et al. demonestrated that mitochondrial microRNAs (mitomiRs) regulate redox balance, apoptosis, mitophagy, calcium handling, and mitochondrial DNA (mtDNA) transcription—an epigenetic layer that may explain why the identical hemodynamic or inflammatory stressor yields different cellular fates with age. Extending this axis into vascular smooth muscle, Rothwell et al. showed that loss-of-function variants of POLG-encoded mitochondrial DNA polymerase (POLG), observed in hypertensive patients, reduce mtDNA copy number and, importantly, trigger secretion of a diffusible mitogen that accelerates proliferation in neighboring cells. They also showed that pharmacological probes such as wedelolactone and MitoTEMPOL could temper this mitogenic drive. This “mitochondria-to-mitogen” mechanism offers a structural path to drug-resistant hypertension: even modest mitochondrial impairment can broadcast a growth signal that remodels the arterial wall.

Vascular aging is inseparable from neurovascular aging. Two original studies quantify how systemic or traumatic perturbations imprint cerebrovascular structure and glial interfaces. A study by Curry-Koski et al. demonstrate that a well-established Marfan syndrome mouse model carrying a heterozygous missense mutation in the gene encoding for fibrillin-1 protein exhibits reduced hippocampal microvascular density, increased blood–brain barrier permeability, and microglial activation at six months—mirroring phenotypes that typically emerge later—supporting a premature neurovascular aging trajectory in connective-tissue disease. Complementary mapping of astrocyte–vessel interactions after diffuse traumatic brain injury by Sabetta et al. reveal sex-dependent, long-lived alterations in Glial Fibrillary Acidic Protein (GFAP)-positive processes contacting cortical vessels, patterns consistent with lingering barrier vulnerability and a cellular substrate for accelerated brain aging after injury. These data extend the focus beyond parenchymal pathology to the perivascular unit, reframing long-term cognitive risk as, in part, a vascular remodeling disease.

At the population interface, new analyses of sleep and post-viral states anchor everyday exposures to measurable vascular aging. Martínez-García et al. report that in healthy adults, shorter sleep duration associates inversely with advanced glycation end-products measured by skin autofluorescence—an accessible marker of cumulative metabolic stress—even after adjustment, reinforcing insufficient sleep as a modifiable vascular risk exposure. Complementing this, Gómez-Sánchez et al. outline a prospective, multimodal protocol incorporating—vascular structure and function, endothelial biomarkers, physical activity, cognition, and mental health—to quantify persistent COVID (“long COVID”) as a vascular-aging condition; notably, it integrates cfPWV/baPWV and the cardio–ankle vascular index with endothelial injury readouts in a 300-participant cohort. Together, these studies illustrate how behaviors and post-viral syndromes may accelerate the arterial age clock, and how acceleration can be quantified reproducibly in real-world settings.

A field-level bibliometric map by Chen et al. highlights that scientific finding itself follows an “aging” pattern. Reviewing more than 1,600 articles at the interface of metabolomics and arterial stiffness, the report shows mature attention clustering around fatty-acid pathways (including eicosatetraenoic acid and arachidonic acid), while bile-acid and microbiome-derived metabolites remain relatively underexplored. Most striking is the underrepresentation of early vascular aging cohorts in youth that warrant more studies and attention, if prevention is to remain a central focus.

Taken together, three distinct themes emerge that integrate these diverse studies into a coherent framework: 1. *endothelial centrality:* from progerin-distorted shear responses to LOX-1–mediated oxidized-lipid effects, the endothelium emerges as a convergence point where mechanical and chemical stressors meet. Restoring “healthy shear” signaling—whether by correcting upstream genome architecture or blocking maladaptive receptors—is a credible anti-aging strategy; 2. *mitochondrial control points:* mitomiRs and POLG dysfunction demonstrate how mitochondrial information flow sets the tone of cellular stress responses and can propagate outward as paracrine growth cues that stiffen vessels; targeting mitochondrial redox and transcriptional crosstalk may yield outsized structural benefits; and 3. *vulnerability of the neurovascular unit:* premature aging in Marfan cerebrovasculature and sex-dependent astrocyte–vessel remodeling after traumatic brain injury position the perivascular niche as a long-lived “memory” of systemic insults, with implications for dementia risk stratification and sex-tailored interventions.

## Prospective

These findings bring several priorities to our attention. In the field of vascular medicine, there is an emergent need to re-center on early vascular aging with youth and mid-life cohorts, combining pulse-wave–based measurements with endothelial injury panels and miRNA/mitomiR signatures to define targetable endpoints before wall stiffness is developed. We should also consider utilizing mechanotransduction therapeutics by translating genotype to flow-response insights into screens for compounds that restore shear-responsive transcripts (for example, suppressing maladaptive galectins) and by testing LOX-1 inhibitors in sex- and age-stratified designs aligned with *ex vivo* aorta data. Another innovative idea is to target mitochondria not only as power plants, but also as important information hubs, by developing mitomiR-guided interventions and evaluating anti-mitogenic, mito-active agents in structural hypertension models to interrupt smooth-muscle remodeling loops initiated by mitochondrial DNA stress. We should embed brain outcomes in vascular trials—adding barrier permeability and astrocyte–vessel interaction metrics, via advanced MRI and blood biomarkers, with sex as an important cofounder. It is also of great importance to give more credits to modifiable exposures such as sleep and infectious diseases, the same way we pay attention to diet and physical activity in the context of preventive medicine, understanding their contribution and impact on vascular function and structure over time.

Collectively, these reports shift vascular aging research from static descriptors—thickness and stiffness—to dynamic, multi-scale pathophysiology that we can measure, modulate, and, crucially, modify early. By integrating mechanistic insights into endothelial shear programs, mitochondrial signaling, and lipid metabolism with translational models of progeria, Marfan syndrome, and traumatic brain injury—and grounding these findings in population-level factors such as sleep and post-COVID phenotyping—this Research Topic charts a roadmap to vascular rejuvenation that is both scientifically ambitious and clinically practical. Our thanks to the authors and reviewers for pushing the field toward that integrated future.

